# Application of Advanced Light Microscopy to the Study of HIV and Its Interactions with the Host

**DOI:** 10.3390/v13020223

**Published:** 2021-02-01

**Authors:** Saveez Saffarian

**Affiliations:** 1 Center for Cell and Genome Science, University of Utah, Salt Lake City, UT 84112, USA; Saffarian@physics.utah.edu; 2 Department of Physics and Astronomy, University of Utah, Salt Lake City, UT 84112, USA; 3 School of Biological Sciences, University of Utah, Salt Lake City, UT 84112, USA

**Keywords:** HIV, iPALM, PALM, STORM, STED, TIRF, Gag, ESCRTs

## Abstract

This review highlights the significant observations of human immunodeficiency virus (HIV) assembly, release and maturation made possible with advanced light microscopy techniques. The advances in technology which now enables these light microscopy measurements are discussed with special emphasis on live imaging approaches including Total Internal Reflection Fluorescence (TIRF), high-resolution light microscopy techniques including PALM and STORM and single molecule measurements, including Fluorescence Resonance Energy Transfer (FRET). The review concludes with a discussion on what new insights and understanding can be expected from these measurements.

## 1. Philosophical Discord

As Arthur Kornberg famously declared “Depend on viruses to open windows” [1], it is therefore not surprising that in the most fundamental quest for understanding Biology, viruses have played a fundamental role and inspired the last two generation of scientists. After the biochemical revolution of the Kornberg’s generation, structural Biology found its footing in viral structures. The amazing symmetry of these structures [2] allowed structural Biology to refine its toolkit and become fundamental in understanding Biological processes [3]. While it is rewarding to focus on our success, it is important to conceptualize the challenge. In the writer’s opinion, the most fundamental challenge is the failure of human brain to understand dynamics of any system with more than three simultaneously interacting parts, which covers almost all Biological processes. This frustration is not new, I would argue it is the fundamental challenge which drove Ramón Cajal to urge his acolytes to focus primarily on reproducibility as the fundamental goal of their scientific endeavor while avoiding creating theories [4]. Are we finally advanced enough to understand the full impact of all molecular dynamics on biological systems? Can viruses lead the way?

This review is primarily focused on the advanced imaging methods for study of human immunodeficiency virus (HIV) and the above questions are too fundamental to be addressed here, nevertheless, given the depth of HIV literature, at the end of the review, a discussion as to how advanced microscopy can be helpful in developing a more complete understanding of complex biological dynamics will be presented.

## 2. HIV Assembly, Budding, and Maturation

Immature HIV virions assemble on the inner leaflet of the plasma membrane, during assembly, two copies of the RNA genome, ~2000 copies of Gag and ~100 copies of Gag-Pol, along with HIV accessory proteins are packaged into the immature virions whose membrane is also decorated with HIV Env proteins [5]. The release of these immature virions from the cell (~140 nm in diameter) is catalyzed by Endosomal Sorting Complexes Required for Transport (ESCRT) [6,7,8,9]. After release, the HIV virions undergo a process of maturation catalyzed by the HIV protease [10,11,12,13], a delay in release of the immature virions results in an untimely activation of the protease and release of non-infectious particles [14]. Many molecular interactions within the immature as well as mature capsid of HIV have been mapped out [15,16,17,18]. We will here focus on the advanced imaging techniques which have been utilized to observe the dynamics by which these molecules come together to form the immature HIV virions and lead to its maturation.

## 3. Total Internal Reflection Fluorescence (TIRF) Microscopy Measurements of HIV Virion Assembly

Originally developed by Daniel Axelrod in the 1980s for observation of cellular membranes [19,20], Total Internal Reflection Fluorescence (TIRF) Microscopy has played a crucial role in understanding the dynamics of HIV assembly and budding. HIV assembly was observed on the plasma membrane of live cells using TIRF microscopy, both through tagging the HIV Gag at its C-terminus with GFP and observing the assembly dynamics by monitoring formation of virus like particles [21] or tracking the assembly of virions with GFP inserted in between the MA and CA domains of Gag [22,23], both of which reported stochastic spontaneous nucleation of Gag on the plasma membrane followed by assembly of virions through addition of Gag molecules following a sigmoidal curve lasting 5–10 min. Using TIRF microscopy as well as photo-switching it was also possible to confirm that new HIV Gag molecules arrive at the assembly site through cytosol while human T-lymphotropic virus (HTLV) Gag assembles by recruiting additional Gag molecules from the adjacent plasma membrane [24]. The evanescent filed generated in TIRF mode can be well calibrated and used as a tool to measure the distance molecules travel away/towards the glass/media interface, this aspect of the TIRF microscopy was significantly developed during study of endocytosis [25,26] and applied to study the assembly of HIV demonstrating the coupling of curvature creation and Gag polymerization during HIV assembly [27].

## 4. Two Color TIRF Microscopy and Timing of Recruitment of Co-Factors

It is needless to say that a major advantage of fluorescent microscopy is the specificity in which cellular components (proteins, lipids, sugars, or nucleic acids) could be linked to a particular fluorescent molecule and therefore be identified spectrally using the fluorescence emission [28]. One of the major advantages of live imaging is therefore simultaneous imaging of multiple components. Although it is possible to separate fluorescence from many fluorescent proteins simultaneously using interlaced pulsed excitation and lifetime gated detection [29,30], these methods require time resolved detection which are impractical for wide field imaging. The emission spectra of fluorescent proteins therefore set a practical limit on the number of proteins which can be identified simultaneously. Given that the emission spectra are typically between 50–75 nm wide and the fluorescent molecules can be chosen with emissions between 500–700 nm, this yields 2 and or 3 fluorescence channels that can be spectrally resolved [31].

### 4.1. Following HIV Genome

Having characterized the Gag recruitment during the assembly of HIV virions, it became possible to use the Gag signal as a marker to time the arrival of different viral components including the RNA genome and various ESCRT components with respect to the stages of assembly. Tracking of mRNA in cytosol became possible by the introduction of a bacteriophage MS2 binding sites into the mRNA sequence while expressing MS2-eGFP in the cells [32,33,34]. Using this technology and two color TIRF, an MS2 tagged partial genome of HIV was tracked during the assembly of the Gag VLPs in live cells and it was shown that the genome arrives very early during the assembly process [35] using similar technology, the genomic RNA was also tracked during its egress from the nucleus [36]. While these experiments are very informative, they are only possible due to the incorporation of a large number (~24) MS2 binding sites into the genomic RNA, whose effects on localization should be considered.

### 4.2. Recruitment of ESCRTs

The timing of arrival of ESCRT components is crucial for understanding HIV release. However, the labeling strategy in fluorescent microscopy is of essential importance. For example, many of the ESCRT proteins act as dominant negative when overexpressed in their GFP fused form [37]. Fluorescently tagged ESCRTs are better tested for their functionality in separate assays before they can be used in live imaging experiments [27,38,39]. Creating cell lines with full replacement of all wild type proteins with fluorescently tagged proteins is also helpful in establishing the functionality of the proteins and in addition allow quantification of number of molecules recruited at any given moment [40,41]. Using these methodologies, the recruitment dynamics of ALIX, TSG101, CHMP4b, and VPS4, all members of ESCRTs have been established with respect to Gag assembly dynamics [38,39,40,42,43,44]. Significant challenges, however, remain, these include observation of budding events in more physiologically relevant cell lines and in particular detection of the exact moment of viral release and its corresponding temporal relationship with recruitment of ESCRTs [45]. Further advances in developing new methodologies are required to parse out the exact mechanism by which ESCRT components catalyze the release of infectious HIV virions.

### 4.3. HIV Capsids

Single molecule TIRF microscopy was used to observe the disassembly of HIV mature capsids in vitro. In these experiments virions are immobilized and permeated on glass cover slips and disassembly of the mature capsid is observed by following the fluorescence signal versus time [46]. These experiments created a useful assay to observe the effects of IP6 and other protein–protein interactions on the dynamics of disassembly [17,47].

TIRF microscopy and specially two color TIRF are therefore very practical techniques that can establish the arrival of molecules within a diffraction limited spot on the microscope, in its most common applications however, TIRF microscopy is limited by the resolution of the optical microscopy.

## 5. Review of High-Resolution Optical Microscopy Techniques

The wave nature of light limits the resolution of any optical microscope to within $\frac{\lambda }{2nSin\left(\emptyset \right)}$, in which λ is the wavelength of light, n is the index of refraction, and θ is the collection angle of the lens [28]. For visible light, the resolution of light microscopy is ~250 nm which is much larger than the 140 nm size of HIV virions. High resolution microscopy has been developed to break the diffraction limit and has two general categories, the first are the methods that utilize engineering of excitation and emission paths utilizing non-linear or interferometric effects to increase the resolution, these methods include stimulated emission depletion (STED) [48], 4 Pi microscopy [49], and structured illumination microscopy (SIM) [50]. These methods are advantageous because they resolve all fluorescent molecules within the sample simultaneously. In contrast the second category of super-resolution techniques depend on the stochastic excitation and detection of single molecules within the sample. These methods mainly utilize that light collected from an object is emanating from a single molecule, the point spread function can be fitted and the location of the single molecule can be determined with nanometer accuracy depending on the number of photons registered from the particle [51]. Once it was discovered that florescent proteins could be switched from an “off” state to an “on” state given an activating pulse of laser [52], these two methods got combined to create stochastic localization microscopy techniques which include Photoactivatable Localization Microscopy (PALM) [53] and Stochastic Optical Reconstruction Microscopy (STORM) [54]. While the first incarnations of these methods were mainly improving the resolution within the focal plane of the microscope, a series of technical advances allowed improvements in the axial resolution of techniques including 3D STORM (~50 nm axial resolution) [55] F-PALM (~50 nm axial resolution) [56] and interferometric PALM (iPALM) (~10 nm axial resolution) [57].

## 6. Applications of High-Resolution Optical Imaging in HIV Biogenesis

What are the most interesting informational content that can be derived from high resolution optical imaging? After all, cryotomography achieves far greater resolution than any optical microscope, so why bother? The answer is twofold, one is specificity and the other is dynamics. Since optical high resolution is fluorescence based and fluorescence can be introduced via genetic tags, high resolution optical imaging can be utilized in fixed samples to resolve the relative positions of two sets of molecules. Again, in this context, HIV Gag is the favorite target and for example positions of ESCRT components, ALIX, CHMP4 and VPS4 were measured with respect to HIV Gag localization with nanometer precision using PALM based techniques [40,58,59]. Aside from ESCRTs, STED microscopy was used to measure the localization of ENV proteins on the surface of mature/immature HIV virions [60]. While these experiments are very informative, the dynamics of the detected structures remain unresolved.

As mentioned, high resolution microscopy also has the capability of resolving the dynamics of viral components. This allows tracking of single molecules in and around HIV virions which includes the measurement of diffusion of HIV Gag molecules into the budding HIV Gag VLPs performed using a combination of PALM single molecule tracking and TIRF microscopy [61]. Individual ENV proteins on the surface of budding HIV virions have also been tracked using iPALM microscopy [62], a combination of these tracking algorithms along with identification of molecular interactions in the cytoplasmic tail of ENV and HIV Gag promise a live observation of such interactions [61,62].

One of the fundamental challenges in the study of HIV maturation has always been synchronization, since both assembly, release, and maturation are all stochastic processes, it is hard to parse one event out of the three for detailed measurements in bulk. Single virion assays however promise to overcome this obstacle by focusing on one virion at a time. Utilizing a light deactivated maturation inhibitor [63], STED was used to time the process of re-organization of Gag during maturation whose half-life was measured to take 30 ± 10 min [64]. In principle it is possible to couple these measurements with fast freezing approaches and cryotomography which would provide further details within these structures in coming future.

What molecular interactions and dynamics play a role in activating the HIV protease has been a fundamental question. Recently iPALM microscopy was used to detect dynamics within the immature lattice of HIV [65], it is not clear if these dynamics are necessarily involved in the activation of the protease, however the data is suggesting further biochemical and structural validation coupled with iPALM microscopy will likely answer these questions.

It is therefore fair to conclude that the high-resolution microcopy techniques offer a very strong toolset for the study of HIV virions, further coupling of these techniques with each other and biochemical and structural analysis will likely uncover new biological mechanisms.

## 7. Fluctuation Spectroscopy Techniques

There is likely no other technique which utilizes the dynamic nature of biological processes as well as fluctuation-based measurements. Fluctuation spectroscopy is based on the Onsager principle and fluctuation dissipation theorem [66] and states that all important kinetic rates within a biochemical process can be measured by following the fluctuations within the system. Fluorescence correlation spectroscopy (FCS) pioneered by Elson and Web in 1970s applied this principle to measurements of fluorescence fluctuations and successfully measured kinetics of protein–DNA binding and diffusion [67]. FCS was plagued by the microscope objective design, specifically the non-infinity corrected objectives, therefore Elson and co-workers created Fluorescence Recovery after Photobleaching which later became more known as FRAP to get more relevant cellular data in the 1980s and 1990s [68]. The introduction of infinity corrected objectives and the dawn of single molecule detection revived the FCS as a relevant technique applied to the study of cellular dynamics [69]. In addition to the temporal analysis of the fluorescence fluctuations, analysis of moments of fluorescence revealed that fluctuation can inform both dynamics as well as the brightness of molecules [70,71,72]. Fluctuation spectroscopy can now be applied in raster scanning confocal as well as TIRF to detect cellular connectivity maps as well as diffusion coefficients, brightness distribution of fluorescence molecules in cellular environments [51,73,74,75,76,77,78]. In advanced theoretical analysis, higher order moments of fluctuations can be used to probe out of equilibrium processes which may turn out to be very useful if detected in Biological processes [79,80,81,82,83]. Techniques to measure fluctuations within a system in a parallel fashion are currently under development [84].

## 8. Application of Fluctuation Spectroscopy to HIV Biology

The brightness analysis enabled by fluctuation spectroscopy enabled the measurement of the number of Gag molecules packaged in the immature lattice of HIV virions. At the time, there was significant interest in understanding whether the immature lattice of HIV is fully packed as was early on proposed [85]. FCS measurements were used to detect the diffusion of Gag molecules and Gag–Gag interactions in cytosol [86]. The measurements of brightness on freely diffusing HIV Gag-eGFP VLPs demonstrated that the measured brightness per virion which corresponded to 1000–2000 Gag-eGFP/VLP is far below the expected full packaging of 5000 Gag-eGFP/VLP [87], these measurements were also confirmed by cryotomography [88]. More recently the brightness distribution was used within the cytosol to detect the state of oligomerization of Gag during HIV budding [89]. A combination of STED and scanning FCS was also used to detect the dynamics of PIP2 during early stages of HIV assembly [90,91]. Fluctuation spectroscopy therefore offers both measurements of dynamics as well as status of molecular aggregates which can be very informative for the study of HIV budding and maturation.

## 9. Fluorescence Resonance Energy Transfer (FRET)

When a fluorophore absorbs a photon, the electronic state of the fluorophore transitions to the excited state. In many cased this excited electron decays back to the ground state through generation of heat, and in some cases the electron emits a photon with a slightly lower energy which will be detected as a stoke shifted fluorescence signal [28]. If, however, there exists a fluorophore with a slightly smaller excitation energy gap at very close vicinity of the excited fluorophore, the energy within the excited fluorophore tunnels into the second fluorophore in a process significantly dependent on the two fluorophores distances and dipole orientations (R_0_/R)^6^ in which R_0_ is ~5–10 nm. When the second fluorophore relaxes to its corresponding ground state, a photon is emitted which is characteristic of the florescence from the second fluorophore, this process is called Fluorescence Resonance Energy Transfer (FRET) [28,92]. FRET is a very precise molecular ruler with a range of 3–10 nm. This range makes it ideal for detecting the conformational conformations of macromolecules. It is not therefore surprising that single molecule FRET was developed rapidly to visualize protein and DNA–Protein interactions [93,94,95]. FRET has been used efficiently to detect conformational changes during binding of HIV Gag to membrane [96] as well as conformational dynamics within single HIV ENV proteins [97,98,99]. The observation of these conformational states coupled with their interactions with neutralizing antibodies promises very interesting studies soon.

## 10. Other Significant Methodologies

This review is by no means comprehensive since there are so many create ways to mix and combine advance light imaging experiments to measure significant biological processes. Specifically, the author would like to point out the methodologies developed to visualize viral entry and early infection which are a critical aspect of HIV biology which are mostly reviewed elsewhere [100,101,102,103].

## 11. Outlook and Future Promises

What is the premise and limitation of advanced optical methodologies? Would advance optical imaging have a fundamental impact on our understanding of virus biology, at the same level as biochemistry and structural biology? After all, the significant contribution of biochemistry is evident, since an annual detection of metabolites in blood is the most critical tool in diagnosis of a variety of diseases. In parallel, almost all small molecule inhibitors have tremendously benefitted from structural biology. Can advanced light microscopy ever achieve such physiological relevance?

While the answer to the above question will become naturally clear in the next decades, the fundamental power of advanced light microscopy techniques is in measuring dynamics in biological processes far away from equilibrium. While detailed protein–protein and enzymatic reactions identified by biochemistry and structural biology would always be valid, there is no guarantee that these interactions are playing out as we conceive them in biological processes, after all, all models build by our human brains are limited to keeping focus only on a few reactions at a time. It is therefore at least conceivable that multi-point multi component live imaging approaches, powered by advanced light microscopy techniques, coupled with advances in artificial intelligence, could uncover new pattern dynamics. In principle, these dynamics would not be easily visualized by man maid theoretical models and cannot be deduced from biochemical and structural data alone. Would understanding of these patterns contribute enough to be useful as a diagnostic or medicinal process? The question will remain to be answered in the next few decades.
